# Effectiveness of booster vaccination with inactivated COVID-19 vaccines against SARS-CoV-2 Omicron BA.2 infection in Guangdong, China: a cohort study

**DOI:** 10.3389/fimmu.2023.1257360

**Published:** 2023-10-17

**Authors:** Xiaofeng He, Biao Zeng, Ye Wang, Yulian Pang, Meng Zhang, Ting Hu, Yuanhao Liang, Min Kang, Shixing Tang

**Affiliations:** ^1^ Department of Epidemiology, School of Public Health, Southern Medical University, Guangzhou, China; ^2^ Institute of Evidence-Based Medicine, Heping Hospital Affiliated to Changzhi Medical College, Changzhi, China; ^3^ Institute of Infectious Disease Control and Prevention, Guangdong Provincial Center for Disease Control and Prevention, Guangzhou, China; ^4^ Department of Epidemiology, School of Medicine, Jinan University, Guangzhou, China; ^5^ Department of Infectious Diseases, Nanfang Hospital, Southern Medical University, Guangzhou, China

**Keywords:** COVID-19, inactivated vaccine, vaccine effectiveness, booster vaccination, Omicron variant

## Abstract

The effectiveness of COVID-19 vaccines wanes over time and the emergence of the SARS-CoV-2 Omicron variant led to the accelerated expansion of efforts for booster vaccination. However, the effect and contribution of booster vaccination with inactivated COVID-19 vaccines remain to be evaluated. We conducted a retrospective close contacts cohort study to analyze the epidemiological characteristics and Omicron infection risk, and to evaluate the effectiveness of booster vaccination with inactivated COVID-19 vaccines against SARS-CoV-2 infection, symptomatic COVID-19, and COVID-19 pneumonia during the outbreaks of Omicron BA.2 infection from 1 February to 31 July 2022 in Guangdong, China. A total of 46,547 close contacts were identified while 6.3% contracted Omicron BA.2 infection, 1.8% were asymptomatic infection, 4.1% developed mild COVID-19, and 0.3% had COVID-19 pneumonia. We found that females and individuals aged 0-17 or ≥ 60 years old were more prone to SARS-CoV-2 infection. The vaccinated individuals showed lower infection risk when compared with the unvaccinated people. The effectiveness of booster vaccination with inactivated COVID-19 vaccines against SARS-CoV-2 infection and symptomatic COVID-19 was 28.6% (95% CI: 11.6%, 35.0%) and 39.6% (95% CI: 30.0, 47.9) among adults aged ≥ 18 years old, respectively when compared with full vaccination. Booster vaccination provided a moderate level of protection against SARS-CoV-2 infection (VE: 49.9%, 95% CI: 22.3%-67.7%) and symptomatic COVID-19 (VE: 62.6%, 95% CI: 36.2%-78.0%) among adults aged ≥ 60 years old. Moreover, the effectiveness of booster vaccination was 52.2% (95% CI: 21.3%, 70.9%) and 83.8% (95% CI: 28.1%, 96.3%) against COVID-19 pneumonia in adults aged ≥ 18 and ≥ 60 years old, respectively. The reduction of absolute risk rate of COVID-19 pneumonia in the booster vaccination group was 0·96% (95% CI: 0.33%, 1.11%), and the number needed to vaccinate to prevent one case of COVID-19 pneumonia was 104 (95% CI: 91, 303) in adults aged ≥ 60 years old. In summary, booster vaccination with inactivated COVID-19 vaccines provides a low level of protection against infection and symptomatic in adults of 18-59 years old, and a moderate level of protection in older adults of more than 60 years old, but a high level of protection against COVID-19 pneumonia in older adults.

## Introduction

The Omicron (B.1.1.529) variant quickly became the dominant circulating SARS-CoV-2 variant worldwide because of its enhanced transmissibility and capability of immune evasion since it was first identified in South Africa on 24 November 2021 (1–3). As of 24 May 2023, the newly reported COVID-19 Omicron cases and deaths reached 342,773 and 3,574 in the past week in the world, respectively (4). Since January 2020, China has implemented the strategy of eliminating COVID-19 by adopting prevention measures of strict physical distance, restrictions on border entry, isolation of COVID-19 cases, separation of close contacts, and application of personal protective equipment. Therefore, COVID-19 epidemics have been largely controlled in China although local outbreaks occurred occasionally. However, a major community epidemic of Omicron BA.2 sub-lineage infection began in early January 2022 in Hong Kong, China (5), followed by outbreaks of Omicron infection in Shenzhen and Shanghai in mainland China between middle and late February 2022 and between March and April 2022, respectively (6). Notably, an unexpected nationwide epidemic of SARS-CoV-2 Omicron variant infection swept across mainland China when the above restriction measures were lifted in November 2022 (7).

COVID-19 vaccination is still considered an indispensable part of the long-term management of the SARS-CoV-2 pandemic (8, 9). According to the World Health Organization (WHO), as of 24 May 2023, 65.53% and 31.40% of individuals have received full vaccination or a booster dose of COVID-19 vaccines worldwide, respectively (4). Since early 2021, four inactivated COVID-19 vaccines including CoronaVac (Sinovac), WIV04 (Sinopharm), HB02 (Sinopharm), and BICV (Biokangtai) have been distributed and administered while HB02 and CoronaVac were most frequently used in mainland China (10, 11). Although full vaccination with the two doses of COVID-19 vaccine provided effective protection against symptomatic COVID-19, especially severe COVID-19 (12), the vaccine effectiveness (VE) was rapidly waning over time (13). Therefore, in November 2021, the National Health Commission of China launched a campaign of booster vaccination with a third dose of COVID-19 vaccines for adults aged ≥ 18 years old and at least six months after full vaccination of COVID-19 vaccine in mainland China by using either homologous or heterologous vaccines.

We previously reported the effectiveness of inactivated COVID-19 vaccines against Delta variant infection of SARS-CoV-2 in real-world settings in Guangdong, China (12). However, the real-world evidence about the role of booster vaccination of inactivated COVID-19 vaccines against infection of the Omicron BA.2 variant remains limited. Although several studies have assessed the effectiveness of the booster dose of inactivated COVID-19 vaccines made in China (5, 6, 14–17), these studies only analyzed the absolute VE of the booster dose but did not compare with full vaccination (6, 14–17) except for two studies that reported the relative VE of booster vaccination by comparing with full vaccination (5, 17). One study in Hong Kong assessed the effectiveness of the CoronaVac vaccine against Omicron BA.2 sub-lineage infection and showed extra protection against severe COVID-19 over full vaccination (5). Another study in Guangzhou, China indicated that booster vaccination with inactivated vaccines did not provide any protection against Omicron BA.2 infection when compared with full vaccination (17). As SARS-CoV-2 Omicron variants keep emerging and show resistance to the neutralizing anti-SAR-CoV-2 antibodies, accelerated expansion and administration of booster vaccination of COVID-19 vaccines are ongoing (18–23). In this study, we conducted a retrospective cohort study to analyze the epidemiological characteristics and risk of Omicron BA.2 infection in the close contacts of subjects infected with the Omicron variant, and to assess the effectiveness of booster vaccination with inactivated COVID-19 vaccines when the stringent restriction measures to prevent SARS-CoV-2 infection were still active and all the close contacts were carefully traced and monitored in Guangdong, China from 1 February to 31 July 2022. Therefore, we could precisely assess the real-world effectiveness of booster vaccination with inactivated COVID-19 vaccines based on different outcomes of Omicron BA.2 variant infection (12).

## Methods

A retrospective close contacts cohort study was conducted to analyze the epidemiological characteristics and Omicron infection risk, and to evaluate the effectiveness of booster vaccination with inactivated COVID-19 vaccines against SARS-CoV-2 infection, symptomatic COVID-19, and COVID-19 pneumonia during the epidemic caused by Omicron sub-lineage BA.2 from 1 February to 31 July 2022 in Guangdong, China according to the Strengthening the Reporting of Observational Studies in Epidemiology (STROBE) guideline (**S1 STROBE Checklist**).

### Study population and design

Close contacts of SARS-CoV-2-infected subjects referred to the people who lived in the same family or stayed in the same public places within close proximity without effective protection as COVID-19 cases or SARS-CoV-2 positive asymptomatic persons two days before (24). All the close contacts were traced, compulsorily quarantined, and tested every 2-3 days by reverse transcription polymerase chain reaction (RT-PCR) tests for SARS-CoV-2 to monitor whether they were infected with SARS-CoV-2. Notably, all the secondary SARS-CoV-2 infections were the close contacts of their index cases before they became infected. Therefore, all the individuals infected with SARS-CoV-2 and their close contacts made up a cohort together.

The information on SARS-CoV-2 infection was obtained from the National Notifiable Infectious Diseases Reporting Information System (NNIDRIS). Data on the close contacts were collected by the Field Epidemiology Investigation System of Guangdong Province. In this study, a total of 46,547 close contacts were identified and asked to centrally isolate if they were not infected or transferred to hospitals for further treatment for COVID-19. Detailed sociodemographic information including age, gender, geographical region, occupation, COVID-19 vaccination status, and COVID-19 severity was recorded and available in the NNIDRIS.

### Vaccination status

**Figure 1:**
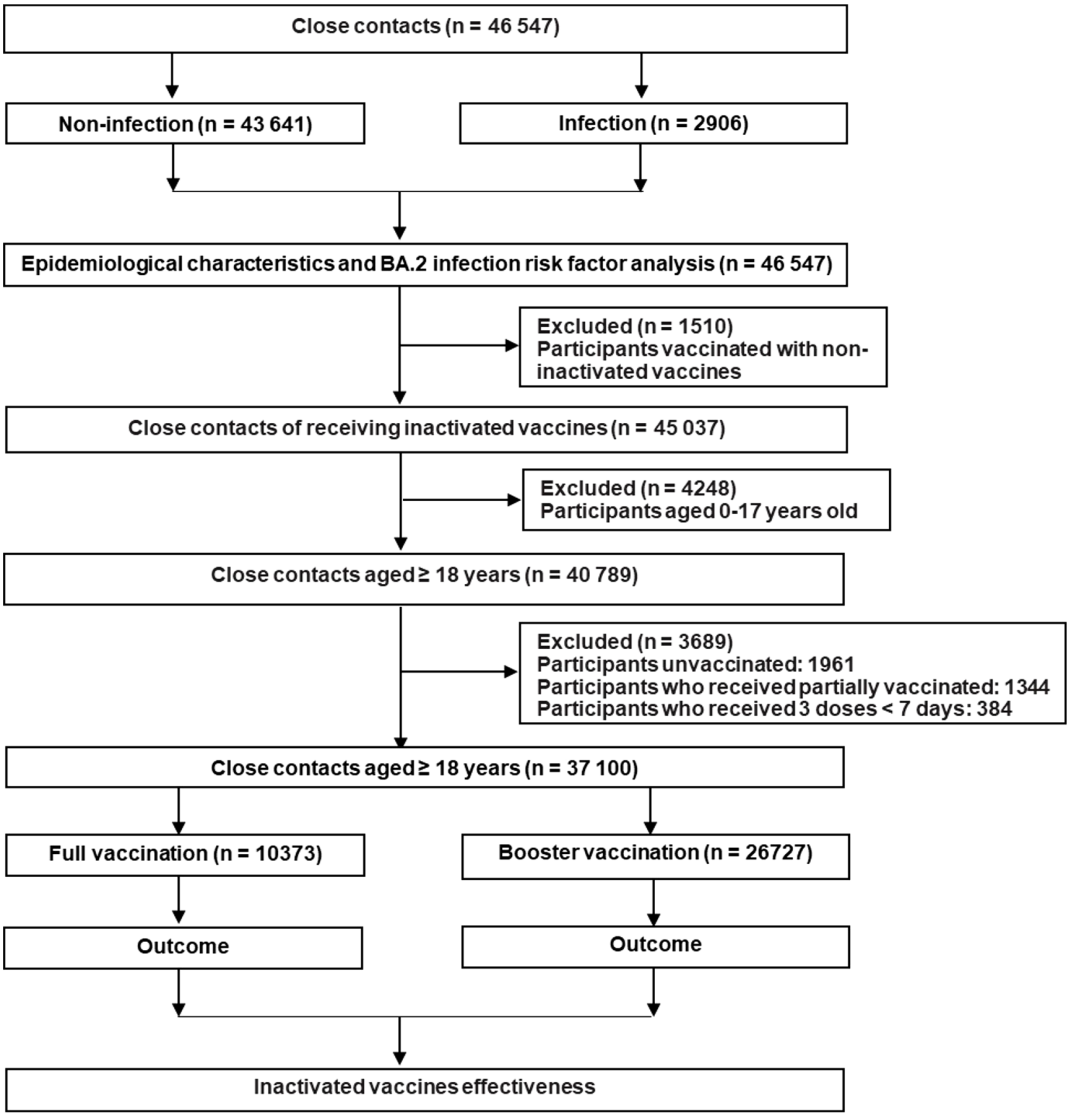
Study design and its flow diagram.

In this study, we classified the close contacts into the unvaccinated group, partially vaccinated group, fully vaccinated group, and booster vaccination group, respectively. The unvaccinated individuals did not receive any COVID-19 vaccine before coming into contact with confirmed COVID-19 cases. Partial vaccination meant ≥ 0 days after the first dose or < 14 days after the second dose of inactivated COVID-19 vaccines. Full vaccination meant ≥ 14 days after the second dose of inactivated COVID-19 vaccines while booster vaccination meant ≥ 7 days after the third dose of inactivated COVID-19 vaccines (25–27). In fact, we excluded the individuals who were not vaccinated or only partially vaccinated because they occupied 4.2% and 2.9% of the total participants, respectively. Moreover, people aged 0–17 years old were also not included in the analysis because they were not covered in the booster immunization program in mainland China. Detailed information about the inclusion and exclusion of the close contacts is presented in **Figure 1**.

### Outcomes

The primary outcome was COVID-19 pneumonia caused by the Omicron BA.2 variant. The secondary outcomes included Omicron infection and symptomatic COVID-19. SARS-CoV-2 infection, symptomatic COVID-19, and COVID-19 pneumonia were diagnosed according to China’s Diagnosis and Treatment Guidelines for COVID-19 Patients (28). Notably, severe COVID-19 was not listed as an outcome since no severe COVID-19 cases were observed in this study.

### Statistical analysis

Baseline characteristics were presented as the number or percentage for the categorical variables or median (inter-quartile range: IQR) for the continuous variables. The categorical and continuous variables were analyzed by Chi-square and Wilcoxon tests, respectively. The cumulative incidence was estimated by dividing the number of new cases of Omicron BA.2 infection by the total number of close contacts analyzed.

The risk factors associated with the occurrence of SARS-CoV-2 Omicron BA.2 infection were evaluated by a stepwise logistic regression model. The VE was first analyzed using the crude VE, i.e. [1–RR]×100% while RR is the rate ratio for the incidence of the defined outcome of SARS-CoV-2 infection. The adjusted VE was calculated using multi-variable ordinary logistic regressions by adjusting the potential confounding factors including age, gender, occupation, interval of vaccination, and geographical regions. Stratified age-related subgroup analyses were performed. Moreover, we also applied the following two measurements to further assess the role or benefits of booster vaccination (1): attributable risk reduction (ARR), which was defined as the arithmetic difference in the cumulative incidence of Omicron BA.2 infection or COVID-19 illness between the booster vaccination group and the full vaccination group, and calculated as ARR = AR_Booster_ – AR_Full_ = AR_full_×adjusted RR – AR_Full_ (2); the number needed to vaccinate (NNV), which was the number needed to vaccinate to prevent one event occurrence and was calculated as the reciprocal of the ARR. All the statistical analysis tests were performed with two-sided tests to determine the corresponding *P* -values. A *P*-value less or equal to 0.05 was considered statistically significant. All data were analyzed using IBM SPSS statistics 25.0.

## Results

### General information of the close contacts

Between 1 February and 31 July 2022, 46,547 close contacts were identified in Guangdong, China (**Table 1**). Among them, 27,065 (58.1%) were male and 19,482 (41.9%) were female. The median age was 34.0 years (IQR: 26.0 ~ 45.0). Moreover, the close contacts were mainly from Shenzhen (35.4%), Guangzhou (29.1%), and Dongguan city (15.8%). Furthermore, 2,747 (5.9%) close contacts did not receive any COVID-19 vaccine while the percentage of partial, full, and booster vaccination with COVID-19 vaccine was 3.9%, 31.7%, and 58.5%, respectively (**Table 1**). Only 4.1% (606/14,741) and 3.7% (1,002/27,233) of adults ≥ 60 years old received full or booster vaccinations , respectively (**Supplementary Figure 1**). Un-vaccinated close contacts were mainly children aged 0-9 years old (23.7%, 652/2,747) while only 1.3% (352/27,233) of the close contacts aged 0-19 years old received booster vaccination probably because children of 0-2 years old were not vaccinated and individuals of 3-17 years old did not receive booster vaccination in mainland China (**Supplementary Figure 1**). Significant differences (*P* < 0.001) were observed regarding gender, age, occupation, and geographical region between SARS-CoV-2 infected and uninfected groups (**Table 1**). To control the potential confounding factor of occupation in affecting the opportunities for vaccination and exposure to SARS-CoV-2, we divided occupations into six categories for further analysis (**Table 1**).

**Table 1:** Characteristics of close contacts in Guangdong, China from February to July, 2022.

Variants	Close contacts (No. [%])			*P value*
	Un-infected (N=43641)	Infected (N=2906)	Total (N=46547)	
Age (years)				
Overall (Median, IQR)	34 (26, 45)	35 (25, 47)	34 (26, 45)	0.776
0-9	2067 (4.7)	263 (9.1)	2330 (5.0)	< 0.001
10-19	2576 (5.9)	230 (7.9)	2806 (6.0)	
20-29	10788 (24.7)	558 (19.2)	11346 (24.4)	
30-39	12736 (29.2)	712 (24.5)	13448 (28.9)	
40-49	8401 (19.3)	535 (18.4)	8936 (19.2)	
50-59	5243 (12.0)	420 (14.4)	5663 (12.2)	
60-69	1290 (3.0)	112 (3.9)	1402 (3.0)	
≥ 70	540 (1.2)	76 (2.6)	616 (1.3)	
Gender				
Male	25556 (58.6)	1509 (51.9)	27065 (58.1)	< 0.001
Female	18085 (41.4)	1397 (48.1)	19482 (41.9)	
Geographical region				
Guangzhou	13031 (29.9)	497 (17.1)	13528 (29.1)	< 0.001
Shenzhen	14955 (34.3)	1520 (52.3)	16475 (35.4)	
Dongguan	6980 (16.0)	379 (13.0)	7359 (15.8)	
Other	8675 (19.8)	510 (17.6)	9185 (19.7)	
Occupation				
Students/Teachers	2061 (4.7)	291 (10.0)	2352 (5.1)	< 0.001
Health care workers	942 (2.2)	43 (1.5)	985 (2.1)	
Restaurant services	321 (0.7)	76 (2.6)	397 (0.8)	
Unemployed/Home	902 (2.1)	677 (23.3)	1579 (3.4)	
Workers	5790 (13.3)	477 (16.4)	6267 (13.5)	
Other	33625 (77.0)	1342 (46.2)	34967 (75.1)	
COVID-19 vaccination status*				
None	2475 (5.7)	272 (9.4)	2747 (5.9)	< 0.001
Partial vaccination	1694 (3.9)	132 (4.5)	1826 (3.9)	
Full vaccination	13677 (31.3)	1064 (36.6)	14741 (31.7)	
Booster vaccination	25795 (59.1)	1438 (49.4)	27233 (58.5)	

*None: not vaccinated; partial vaccination: < 14 days after first dose for viral vector (non-replicating) vaccine, after first dose or < 14 days after second dose for inactivated vaccines, and after first and second dose, or < 14 days after third dose for protein subunit vaccine; full vaccination: ≥ 14 days after first dose for viral vector (non-replicating) vaccine, ≥ 14 days after second dose for inactivated vaccine, ≥ 14 days after third dose for protein subunit vaccine, and < 7 days after booster vaccination; booster vaccination: ≥ 7 days after second dose for viral vector (non-replicating) vaccines or ≥ 7 days after third dose for any COVID-19 vaccine (the second dose was inactivated vaccines).

### Cumulative incidence of Omicron BA.2 infection

Among 46,547 close contacts, 6.2% (95% CI: 6.0%, 6.5%) were confirmed to be infected with Omicron BA.2 sub-lineage with a cumulative incidence of asymptomatic infection, symptomatic COVID-19, mild COVID-19, and COVID-19 pneumonia of 1.8% (95% CI: 1.7%, 2.0%), 4.4% (95% CI: 4.2%, 4.6%), 4.1% (95% CI: 3.9%, 4.2%), and 0.3% (95% CI: 0.3%, 0.4%), respectively (**Table 2**). There were two infection peaks in the groups of 0-9 years old (11.3% [95% CI: 10.1, 12.7%]) and ≥ 60 years old (9.3% [95% CI: 8.1%, 10.7%], **Table 2**, **Supplementary Figure 2**). Furthermore, we found that close contacts who were ≥ 60 years old had the highest incidence (0.7% [95% CI: 0.4%, 1.3%]) of COVID-19 pneumonia (**Table 2**) while the percentage of asymptomatic infection, symptomatic COVID-19, mild COVID-19, and COVID-19 pneumonia was 29.8%, 70.2%, 64.9%, and 5.3%, respectively (**Supplementary Table 1**). However, no deaths were reported in this study (**Supplementary Table 1**). Moreover, we found that the individuals who were unemployed or worked at home had the highest cumulative incidence (42.9% [95% CI: 40.4%, 45.4%]) of BA.2 infection while the cumulative incidence of asymptomatic infection, symptomatic COVID-19, mild COVID-19, and COVID-19 pneumonia was 11.8% (95% CI: 10.3%, 13.5%), 31.1% (95% CI: 28.8%, 33.5%), 28.4% (95% CI: 26.2%, 30.8%), and 2.7% (95% CI: 2.0%, 3.6%), respectively in this population (**Table 2**).

**Table 2:** Incidence of SARS-CoV-2 Omicron BA.2 infection and symptomatic COVID-19 among the close contacts in Guangdong, China, from February to July 2022.

Characteristics	Close contacts	Infection (No. [%])	Infection (No. [%])		Symptomatic COVID-19 (No. [%])	
			Asymptomatic infection	Symptomatic COVID-19	Mild COVID-19	COVID-19 pneumonia
**Overall**	46547	2906 (6.3)	865 (1.9)	2041 (4.4)	1887 (4.1)	154 (0.3)
Age, years						
Median, IQR	34 (26, 45)	35 (25, 47)	38 (28, 49)	34 (23, 46)	33 (23, 45)	40 (29, 50)
0-9	2330	263 (11.3)	54 (2.3)	209 (9.0)	201 (8.6)	8 (0.4)
10-19	2806	230 (8.2)	40 (1.4)	190 (6.8)	184 (6.6)	6 (0.2)
20-29	11346	558 (4.9)	146 (1.3)	412 (3.6)	387 (3.4)	25 (0.2)
30-39	13448	712 (5.3)	226 (1.7)	486 (3.6)	452 (3.4)	34 (0.2)
40-49	8936	535 (6.0)	185 (2.1)	350 (3.9)	311 (3.5)	39 (0.4)
50-59	5663	420 (7.4)	154 (2.7)	266 (4.7)	239 (4.2)	27 (0.5)
≥ 60	2018	188 (9.3)	60 (3.0)	128 (6.3)	113 (5.6)	15 (0.7)
Gender						
Male	27065	1509 (5.6)	460 (1.7)	1049 (3.9)	972 (3.6)	77 (0.3)
Female	19482	1397 (7.2)	405 (2.1)	992 (5.1)	915 (4.7)	77 (0.4)
Geographical region						
Guangzhou	13528	497 (3.7)	47 (0.4)	450 (3.3)	393 (2.9)	57 (0.7)
Shenzhen	16475	1520 (9.2)	377 (2.3)	1143 (6.9)	1070 (6.5)	73 (0.4)
Dongguan	7359	379 (5.2)	260 (3.5)	119 (1.7)	110 (1.5)	9 (0.2)
Other	9185	510 (5.6)	181 (2.0)	329 (3.6)	314 (3.4)	15 (0.2)
Occupation						
Students/Teachers	2352	291 (12.4)	57 (2.4)	234 (10.0)	228 (9.7)	6 (0.3)
Health care workers	985	43 (4.4)	9 (0.9)	34 (3.5)	29 (3.0)	5 (0.5)
Restaurant services	397	76 (19.1)	26 (6.5)	50 (12.6)	43 (10.8)	7 (1.8)
Unemployed/Home	1579	677 (42.9)	186 (11.8)	491 (31.1)	449 (28.4)	42 (2.7)
Workers	6267	477 (7.6)	249 (4.0)	228 (3.6)	208 (3.3)	20 (0.3)
Other	34967	1342 (3.8)	338 (1.0)	1004 (2.8)	930 (2.7)	74 (0.1)
COVID-19 vaccine dose*						
None	2747	272 (9.9)	71 (2.6)	201 (7.3)	181 (6.6)	20 (0.7)
Part vaccination	1826	132 (7.2)	41 (2.2)	91 (5.0)	86 (4.7)	5 (0.3)
Full vaccination	14741	1064 (7.2)	279 (1.9)	785 (5.3)	731 (5.0)	54 (0.3)
Booster vaccination	27233	1438 (5.3)	474 (1.7)	964 (3.6)	889 (3.3)	75 (0.3)

*None: not vaccinated; partial vaccination: < 14 days after first dose for viral vector (non-replicating) vaccine, after first dose or < 14 days after second dose for inactivated vaccines, and after first and second dose, or < 14 days after third dose for protein subunit vaccine; full vaccination: ≥ 14 days after first dose for viral vector (non-replicating) vaccine, ≥ 14 days after second dose for inactivated vaccine, ≥ 14 days after third dose for protein subunit vaccine, and < 7 days after booster vaccination; booster vaccination: ≥ 7 days after second dose for viral vector (non-replicating) vaccines or ≥ 7 days after third dose for any COVID-19 vaccine (the second dose was inactivated vaccines).

**Figure 2:**
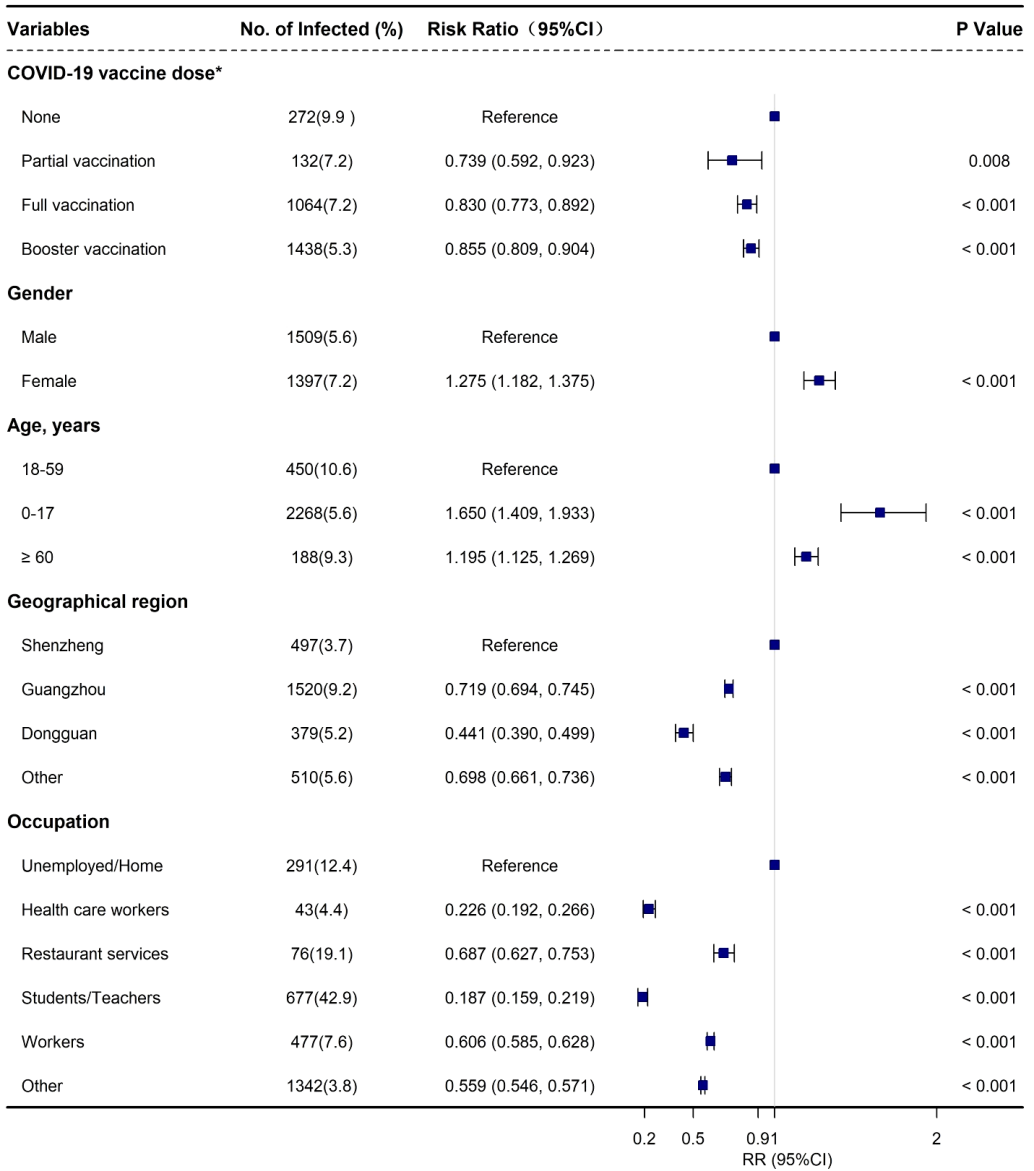
Adjusted risk factors associated with SARS-CoV-2 Omicron BA.2 infection among the close contacts based on logistics regression in Guangdong, China from February to July 2022. *None: not vaccinated; part vaccination: < 14 days after first vaccination for viral vector (non-replicating) vaccine, after first vaccination or < 14 days after second vaccination for COVID-19 inactivated virus vaccine, and after first and second vaccination, or < 14 days after third vaccination COVID-19 protein subunit vaccine (if any); full vaccination: ≥ 14 days after first vaccination for viral vector (non-replicating) vaccine, ≥ 14 days after second vaccination for COVID-19 inactivated virus vaccine, ≥ 14 days after third vaccination for COVID-19 protein subunit vaccine, and < 7 days after booster vaccination (if any); booster vaccination: ≥ 7 days after second dose for COVID-19 viral vector (non-replicating) vaccines or ≥ 7 days after third dose for COVID-19 any vaccine (including protein subunit, inactivated virus, and viral vector [non-replicating] vaccines) (if any).

In addition, females had a slightly higher risk of BA.2 infection with an adjusted ratio risk [aRR] of 1.275 (95% CI: 1.182, 1.375) when compared with males (**Supplementary Table 2**, **Figure 2**). Moreover, an increased risk of infection was associated with age, in particular for the individuals 0-17 years old (aRR: 1.650; 95% CI: 1.409, 1.933) and ≥ 60 years old (aRR: 1.195; 95% CI: 1.125, 1.269) when compared with those aged 18-59 years old (**Supplementary Table 2**, **Figure 2**). Furthermore, a relatively lower risk of infection was observed among the close contacts who received partial vaccination (aRR: 0.739; 95% CI: 0.592, 0.923), full vaccination (aRR: 0.830; 95% CI: 0.773, 0.892), and booster vaccination (aRR: 0.855; 95% CI: 0.809, 0.904), respectively when compared with unvaccinated people (**Supplementary Table 2**, **Figure 2**). We also found that the individuals in Shenzhen had the highest infection incidence followed by Guangzhou and Dongguan (**Supplementary Table 2**, **Figure 2**).

### Effectiveness of booster vaccination

A total of 37,100 close contacts equal to or over 18 years old were included for the evaluation of the effectiveness of inactivated COVID-19 vaccines. Among them, 10,373 (28.0%) were fully vaccinated and 26,727 (72.0%) had received booster vaccination (**Supplementary Table 3**). Significant differences were observed between the two groups regarding age, gender, occupation, and geographical regions (*P* < 0.001, **Supplementary Table 3**). Moreover, the cumulative incidence of the major outcomes, i.e., SARS-COV-2 infection, symptomatic COVID-19, and COVID-19 pneumonia, were significantly different (**Supplementary Table 3**).

**Figure 3:**
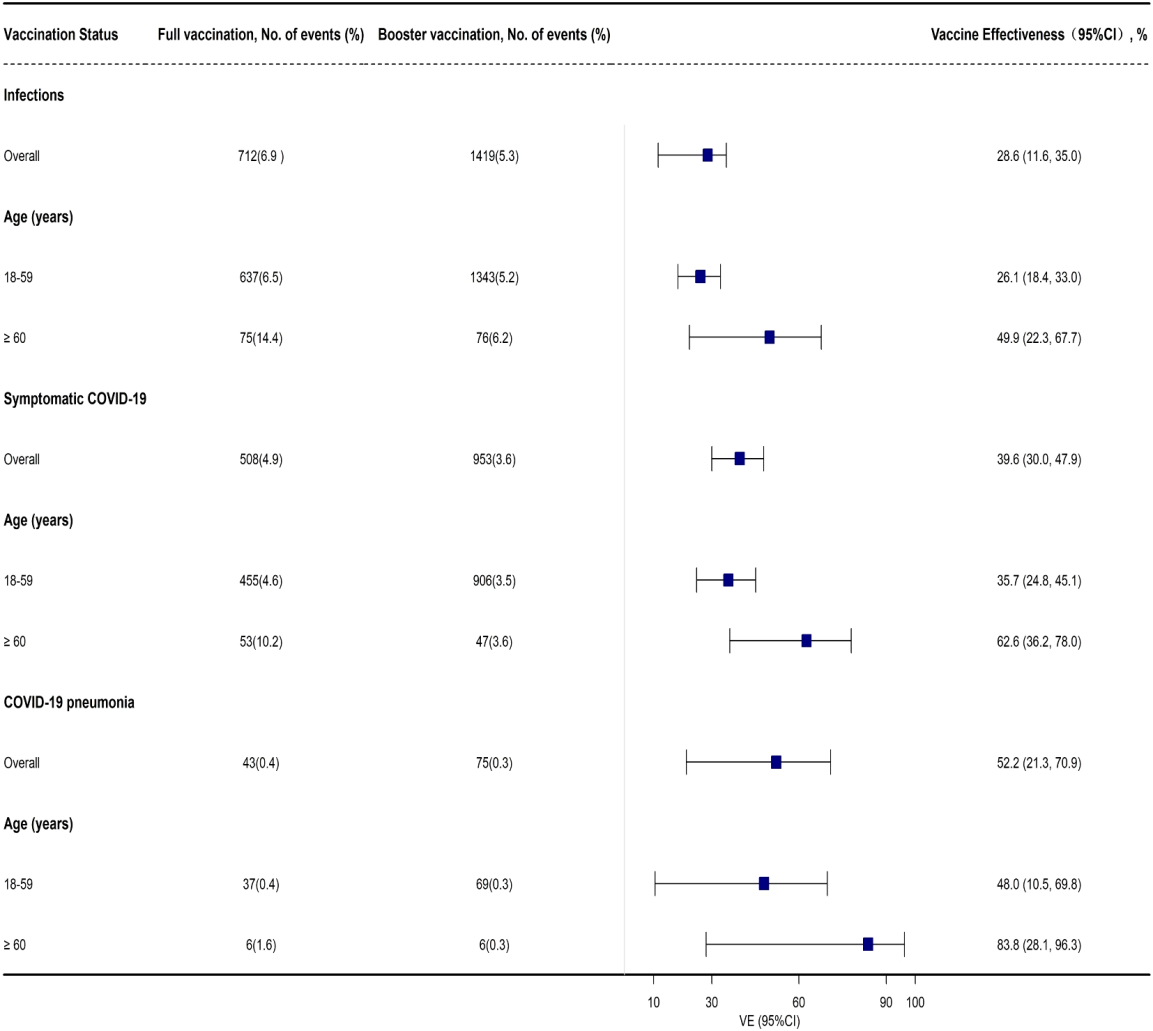
Adjusted effectiveness of booster vaccination of COVID-19 inactivated vaccines against infection and COVID-19 illness cause by SARS-CoV-2 Omicron variant when compared with full vaccination.

Compared with the full vaccination group, the effectiveness of booster vaccination against BA.2 infection was 28.6% (95% CI: 11.6%, 35.0%) among adults ≥ 18 years old. Subgroup analysis indicated that booster vaccination provided a low level of protection against BA.2 infection among adults 18-59 years old with a VE of 26.1% (95% CI: 18.4%, 33.0%) while similar results were also found against symptomatic COVID-19 (**Supplementary Table 4**, **Figure 3**). However, a high level of protection for booster vaccination against COVID-19 pneumonia was found in adults ≥ 60 years old with a VE of 83.8% (95% CI: 28.1%, 96.3%) while only a moderate level of protection was observed to prevent BA.2 infection and symptomatic COVID-19 in this group with a VE of 49.9% (95% CI: 22.3%, 67.7%) and 62.6% (95% CI: 36.2%, 78.0%), respectively (**Supplementary Table 4**, **Figure 3**). However, the effectiveness of booster vaccination to prevent COVID-19 pneumonia was only 52.2% (95% CI: 21.3%, 70.9%) in all the close contacts and 48.0% (95% CI: 10.5%, 69.8%) among the subjects 18-59 years old (**Supplementary Table 4**, **Figure 3**).

### Contribution of booster vaccination to prevent COVID-19 pneumonia

Overall, the ARR for COVID-19 pneumonia contributed by booster vaccination was 0.22% (95% CI: 0.09%, 0.30%), and the NNV to prevent one case of COVID-19 pneumonia was 455 (95% CI: 333–1,111) for all the participants analyzed (**Table 3**). Among the adults 18-59 years old, the corresponding ARR was 0.18% (95% CI: 0.04%, 0.26%), and the NNV was 556 (95% CI: 385, 2,500; **Table 3**). Among adults ≥ 60 years old, the ARR increased to 0.96% (95% CI: 0.33%, 1.11%), and the NNV was only 104 (95% CI: 91, 303; **Table 3**).

**Table 3:** Contribution of booster vaccination of inactivated COVID-19 vaccines to prevent COVID-19 pneumonia.

SARS-CoV-2 Omicron BA.2 variant	Event rate in full vaccination (n/N)	Adjusted RR (95% CI)	Adjusted event rate in booster vaccination (95% CI)	Adjusted ARR (95% CI)	NNV (95% CI)
COVID-19 pneumonia					
Overall	43/10373 (0.42%)	0.478 (0.291, 0.787)	0.20% (0.12, 0.33)	0.22% (0.09, 0.30)	455 (333, 1111)
Age (years)					
18-59	37/9852 (0.38%)	0.520 (0.302, 0.895)	0.20% (0.12, 0.34)	0.18% (0.04, 0.26)	556 (385, 2500)
≥ 60	6/521 (1.15%)	0.162 (0.037, 0.709)	0.19% (0.043, 0.82)	0.96% (0.33, 1.11)	104 (91, 303)

RR, risk ratio; ARR, absolute risk reduction; NNV, Number needed to vaccinate.

## Discussion

We conducted a real-world retrospective cohort study to analyze the epidemiological characteristics of SARS-CoV-2 Omicron BA.2 infection and the risk factors among 46,547 close contacts of COVID-19 patients, and to evaluate the effectiveness of booster vaccination with inactivated COVID-19 vaccines against infection or COVID-19 pneumonia caused by Omicron BA.2 sub-lineage by comparing with full vaccination group in Guangdong, China.

The stringent prevention strategy and measures worked well and significantly inhibited the transmission and expansion of SARS-CoV-2 infection in China, which in turn may explain the overall low cumulative incidence (6.2%) of Omicron variant infection among the close contacts of SARS-CoV-2 infected subjects in our study. A similar study conducted in 2021 when the SARS-CoV-2 Delta variant epidemic occurred in Guangdong province, China, indicated a very low incidence of 1.3% of Delta infection among 10,805 close contacts (12) partly because the early diagnosis of SARS-CoV-2-infected index cases and prompt identification, tracing, and isolation of their close contacts limited its further transmission in this population. Furthermore, in the current study, the percentage of symptomatic COVID-19, mild COVID-19, and COVID-19 pneumonia was 70.2%, 64.9%, and 5.3% among the Omicron-infected cases, respectively, which was significantly lower than the corresponding rates (97.8%, 88.2%, and 14.0%, respectively) among the Delta-infected cases (12). The two studies in the same population showed a higher incidence of Omicron infection, but a lower percentage of COVID-19 pneumonia caused by the Omicron variant compared to the Delta variant, which supported the greater transmissibility but lower virulence of the Omicron variant compared to the Delta variant (29–32). Indeed, previous studies have confirmed lower severity in the cases of Omicron infection when compared with various waves of SARS-CoV-2 infections (33–38).

In this study, several risk factors were associated with infection with the Omicron variant, i.e. occupation, gender, and vaccination status. Specifically, those unemployed or subjects who worked at home and females showed higher infection risk probably because they had more opportunities for household contact with SARS-CoV-2-infected subjects since previous studies identified household contact as the main route of SARS-CoV-2 transmission due to more frequent and longer unprotected exposure to SARS-CoV-2 (39, 40). Interestingly, we found that COVID-19 vaccination, even partial vaccination, resulted in a relatively lower incidence of Omicron infection when compared with the un-vaccinated subjects. However, it may not mean that COVID-19 vaccines can prevent Omicron infection since the effectiveness of COVID-19 vaccines was lower than 50%, which is the lower limit of the vaccine effectiveness set by WHO (41). In this study, we did not adjust the infection risk by considering all the confounding factors. Therefore, the relatively lower incidence of Omicron infection in the vaccinated group may be affected by other unmeasured factors, and further evaluation is needed.

An important goal of the current study was to evaluate the role of booster vaccination with inactivated COVID-19 vaccines to prevent infection caused by the Omicron variant in real-world settings. Our results confirmed that booster vaccination could only provide a low level of protection against Omicron infection and symptomatic COVID-19 in adults 18-59 years, but a high level of protection against COVID-19 pneumonia in adults ≥ 60 years old when compared with full vaccination. To the best of our knowledge, there were only two published studies that reported the relative effectiveness of booster vaccination with inactivated COVID-19 vaccines against infection caused by the Omicron variant (5, 17). In a test-negative case-control study in Guangzhou, China, the booster dose of inactivated COVID-19 vaccines did not provide any protection against Omicron infection when compared with full vaccination (17). A study in Hong Kong showed that the effectiveness of booster vaccination against mild or moderate COVID-19 caused by the Omicron BA.2 variant was 35·7% and 46.9% among the subjects aged 20-59 and ≥ 60 years old, respectively (5). However, booster vaccination is critical for preventing severe or fatal COVID-19 disease caused by Omicron BA.2 with an effectiveness of 80% for those aged more than 20 years old (5). Our results were consistent with the findings about the role of booster vaccination with other types of COVID-19 vaccines against Omicron BA.2 infection or COVID-19 (5, 42–44). All these studies confirmed the importance of booster vaccination with COVID-19 vaccines to mitigate the severity of COVID-19, especially in old adults, which is consistent with the WHO recommendation about the administration of COVID-19 vaccines for this group (45).

In the current study, the benefit of booster vaccination in old adults was further confirmed by the bigger reduction of attributable risk rate and smaller number needed to vaccinate (NNV). We found that booster vaccination reduced the incidence of COVID-19 pneumonia by 0.96%, and the NNV to prevent one COVID-19 pneumonia case was 104 in adults ≥ 60 years old. However, in adults 18-59 years old, the ARR was only 0.18%, and the NNV was as many as 556. There are five-fold differences in the ARR and NNV between adults over 60 years old and 18-59 years old. In other words, booster vaccination with inactivated COVID-19 vaccines may decrease the incidence of COVID-19 pneumonia and save the medical cost by about five-fold over the full vaccination group. Considering the fact that COVID-19 vaccines are more effective in reducing the occurrence of severe COVID-19 and COVID-19-related mortality in old adults, booster vaccination with COVID-19 vaccines is presumably more cost-effective in mitigating severe COVID-19 in old people. This remains to be elucidated through comprehensive cost-effectiveness analysis.

Our study had several limitations. First, our study was conducted before the stringent prevention measures were lifted at the end of 2022 in China. All the close contacts of SARS-CoV-2 positive cases were immediately identified and quarantined. Therefore, the results cannot be expanded directly to situations where no specific prevention measures exist and people can move and communicate freely. However, the effectiveness of the booster vaccination we observed is still useful for policy making and the administration of COVID-19 vaccines. Second, unmeasured confounders might compromise the validity of our results although we had tried to control the known covariates. Third, we did not use hospitalization as an outcome to evaluate vaccine effectiveness because all SARS-CoV-2- positive people must be isolated and hospitalized in China regardless of the severity of COVID-19. Instead, we utilized symptomatic COVID-19 or COVID-19 pneumonia as the major outcome of SARS-CoV-2 infection to evaluate the overall effectiveness of the COVID-19 vaccines, especially in mitigating disease severity of COVID-19 because Omicron BA. 2 infection is usually less severe than Delta variant (38, 46). Fourth, the possible differences between inactivated COVID-19 vaccines from different vendors and the duration after full or booster vaccination were not analyzed in our study due to the short period of observation. Fifth, we confirmed that senior adults over 60 years old as the best target population to receive a booster vaccination. However, they accounted for 4.3% of the close contacts analyzed in our study. The relatively small sample size may affect the reliability of our results. Finally, we did not detect anti-SARS-CoV-2 antibodies or evaluate the role of hybrid immunity. Therefore, the underlying mechanism of booster vaccination to mitigate the severity of COVID-19 was not clear.

In summary, our findings indicate that booster vaccination with inactivated COVID-19 vaccines is necessary and useful in mitigating the severity of COVID-19 caused by the SARS-CoV-2 Omicron variant. The target population should be adults over 60 years old. Further investigation is definitely needed about the durability of booster vaccination and the effectiveness of COVID-19 vaccines in preventing the emerging Omicron variants with enhanced capability for immune evasion.

## Data availability statement

The original contributions presented in the study are included in the article/**Supplementary Material**. Further inquiries can be directed to the corresponding authors.

## Ethics statement

The studies involving human participants were reviewed and approved by the China CDC Ethical Review Committee (approval number 202210). Written informed consent was waived because the data in the study were collected from administrative requirements of disease control and surveillance by Guangdong Provincial Center for Disease Control and Prevention as required by the National Health Commission. Analytical data sets were de-linked and anonymized in this study.

## Author contributions

XH: Conceptualization, Project administration, Writing – original draft, Writing – review & editing, Data curation, Formal Analysis, Methodology, Validation. BZ: Conceptualization, Data curation, Project administration, Writing – original draft, Writing – review & editing, Investigation. YW: Data curation, Investigation, Writing – review & editing. YP: Data curation, Investigation, Writing – review & editing. MZ: Investigation, Writing – review & editing. TH: Investigation, Writing – review & editing. YL: Writing – review & editing, Formal Analysis. MK: Writing – review & editing, Conceptualization, Project administration, Supervision, Writing – original draft. ST: Conceptualization, Project administration, Supervision, Writing – original draft, Writing – review & editing.
