# Supplementary material for: Effectiveness of booster vaccination with inactivated COVID-19 vaccines against SARS-CoV-2 Omicron BA.2 infection in Guangdong, China: a cohort study
Source: Front Immunol. 2023 Oct 17;14:1257360. doi: 10.3389/fimmu.2023.1257360 (PMC10616523; doi:10.3389/fimmu.2023.1257360)
Supplement: Supplementary file 2 [file DataSheet_2.docx]

**Supplementary Figure 1** Vaccination rate of close contacts by age and COVID-19 vaccination status
