# Supplementary material for: Effectiveness of booster vaccination with inactivated COVID-19 vaccines against SARS-CoV-2 Omicron BA.2 infection in Guangdong, China: a cohort study
Source: Front Immunol. 2023 Oct 17;14:1257360. doi: 10.3389/fimmu.2023.1257360 (PMC10616523; doi:10.3389/fimmu.2023.1257360)
Supplement: Supplementary file 4 [file DataSheet_4.docx]

**Supplementary Table 1** Characteristics of the SARS-CoV-2 Omicron BA.2 infectors of close contacts according to infection type, Guangdong, China, February–July 2022

| **Characteristics of infectors of close contacts** | **SARS-CoV-2 infection (No. [%])** | | **Symptomatic COVID-19 (No. [%])** | | **Overall**  **(N = 2906)** |
| --- | --- | --- | --- | --- | --- |
|  | **Asymptomatic infection**  **(N = 865)** | **Symptomatic COVID-19**  **(N = 2041)** | **Mild COVID-19 (N = 1887)** | **COVID-19 pneumonia**  **(N = 154)** |  |
| **Age, years** | | | | | |
| Median, IQR | 38.0 (28.0, 49.0) | 34.0 (23.0, 46.0) | 33.0 (23.0, 45.0) | 40.0 (29.0, 50.0) | 35.0 (25.0, 47.0) |
| 0-9 | 54 (6.2) | 209 (10.2) | 201 (10.6) | 8 (5.2) | 263 (9.1) |
| 10-19 | 40 (4.6) | 190 (9.3) | 184 (9.7) | 6 (3.9) | 230 (7.9) |
| 20-29 | 146 (16.9) | 412 (20.2) | 387 (20.5) | 25 (16.2) | 558 (19.2) |
| 30-39 | 226 (26.1) | 486 (23.8) | 452 (24.0) | 34 (22.1) | 712 (24.5) |
| 40-49 | 185 (21.4) | 350 (17.2) | 311 (16.5) | 39 (25.3) | 535 (18.4) |
| 50-59 | 154 (17.8) | 266 (13.0) | 239 (12.7) | 27 (17.5) | 420 (14.4) |
| 60-69 | 32 (3.7) | 80 (3.9) | 74 (3.9) | 6 (3.9) | 112 (3.9) |
| ≥ 70 | 28 (3.3) | 48 (2.4) | 39 (2.1) | 9 (5.8) | 76 (2.6) |
| **Gender** | | | | | |
| Male | 460 (53.2) | 1049 (51.4) | 972 (51.5) | 77 (50.0) | 1509 (51.9) |
| Female | 405 (46.8) | 992 (48.6) | 915 (48.5) | 77 (50.0) | 1397 (48.1) |
| **Geographical region** | | | | | |
| Guangzhou | 47 (5.4) | 450 (22.1) | 393 (20.8) | 57 (37.0) | 497 (17.1) |
| Shenzhen | 377 (43.6) | 1143 (56.0) | 1070 (56.7) | 73 (47.4) | 1520 (52.3) |
| Dongguan | 260 (30.1) | 119 (5.8) | 110 (5.8) | 9 (5.8) | 379 (13.0) |
| Other | 181 (20.9) | 329 (16.1) | 314 (16.6) | 15 (9.8) | 510 (17.6) |
| **Occupation** | | | | | |
| Students/Teachers | 57 (6.6) | 234 (11.5) | 228 (12.1) | 6 (3.9) | 291 (10.0) |
| Health care workers | 9 (1.0) | 34 (1.7) | 29 (1.5) | 5 (3.2) | 43 (1.5) |
| Restaurant services | 26 (3.0) | 50 (2.4) | 43 (2.3) | 7 (4.5) | 76 (2.6) |
| Unemployed/Home | 186 (21.5) | 491 (24.0) | 449 (23.8) | 42 (27.3) | 677 (23.3) |
| Workers | 249 (28.8) | 228 (11.2) | 208 (11.0) | 20 (13.0) | 477 (16.4) |
| Other | 338 (39.1) | 1004 (49.2) | 930 (49.3) | 74 (48.1) | 1342 (46.2) |
| **COVID-19 vaccine dose*** | | | | | |
| None | 71 (8.2) | 201 (9.8) | 181 (9.6) | 20 (13.0) | 272 (9.4) |
| Partial vaccination | 41 (4.7) | 91 (4.5) | 86 (4.6) | 5 (3.2) | 132 (4.5) |
| Full vaccination | 279 (32.3) | 785 (38.5) | 731 (38.7) | 54 (35.7) | 1064 (37.0) |
| Booster vaccination | 474 (54.8) | 964 (47.2) | 889 (47.1) | 75 (48.1) | 1438 (49.1) |
| **Number of comorbidities** | | | | | |
| 0 | 754 (87.2) | 856 (82.2) | 723 (81.5) | 133 (86.4) | 2610 (89.8) |
| ≥ 1 | 111 (12.8) | 185 (17.8) | 164 (18.5) | 21 (13.6) | 296 (10.2) |
| **Ct value** | | | | | |
| ORF1ab | 26.1 (21.2, 32.0) | 25.0 (20.2, 30.3) | 25.0 (20.0, 30.1) | 26.2 (21.2, 31.7) | 25.5 (20.7, 31.0) |
| N gene | 26.0 (20.3, 31.0) | 24.0 (19.0, 30.0) | 24.0 (19.0, 29.7) | 25.0 (20.4, 31.0) | 24.6 (19.6, 30.0) |

*None: not vaccinated; partial vaccination: < 14 days after first vaccination for viral vector (non-replicating) vaccine, after first vaccination or < 14 days after second vaccination for COVID-19 inactivated virus vaccine, and after first and second vaccination, or < 14 days after third vaccination COVID-19 protein subunit vaccine (if any); full vaccination: ≥ 14 days after first vaccination for viral vector (non-replicating) vaccine, ≥ 14 days after second vaccination for COVID-19 inactivated virus vaccine, ≥ 14 days after third vaccination for COVID-19 protein subunit vaccine, and < 7 days after booster vaccination (if any); booster vaccination: ≥ 7 days after second dose for COVID-19 viral vector (non-replicating) vaccines or ≥ 7 days after third dose for COVID-19 any vaccine (including protein subunit, inactivated virus, and viral vector [non-replicating] vaccines) (if any).
