# Supplementary material for: Effectiveness of booster vaccination with inactivated COVID-19 vaccines against SARS-CoV-2 Omicron BA.2 infection in Guangdong, China: a cohort study
Source: Front Immunol. 2023 Oct 17;14:1257360. doi: 10.3389/fimmu.2023.1257360 (PMC10616523; doi:10.3389/fimmu.2023.1257360)
Supplement: Supplementary file 5 [file DataSheet_5.docx]

**Supplementary Table 2** Risk factors associated with SARS-CoV-2 Omicron BA.2 infection among the close contacts based on logistic regression analysis in Guangdong, China from February to July 2022

| **Variables** | **No. of Participants** | **No. of Infected** | **Incidence (% [95% CI])** | **Crude RR (95% CI)** | ***P* value** | **Adjusted RR (95% CI)** | ***P* value** |
| --- | --- | --- | --- | --- | --- | --- | --- |
| **Overall** | 46547 | 2906 | 6.2 (6.0, 6.5) | **–** | – | – | – |
| **COVID-19 vaccine dose*** | | | | | | | |
| None | 2747 | 272 | 9.9 (8.8, 11.1) | Reference | – | – | – |
| Partial vaccination | 1826 | 132 | 7.2 (6.1, 8.5) | 0.709 (0.571, 0.881) | 0.002 | 0.739 (0.592, 0.923) | 0.008 |
| Full vaccination | 14741 | 1064 | 7.2 (6.8, 7.7) | 0.841 (0.784, 0.902) | < 0.001 | 0.830 (0.773, 0.892) | < 0.001 |
| Booster vaccination | 27233 | 1438 | 5.3 (5.0, 5.6) | 0.797 (0.762, 0.834) | < 0.001 | 0.855 (0.809, 0.904) | < 0.001 |
| **Gender** | | | | | | | |
| Male | 27065 | 1509 | 5.6 (5.3, 5.9) | Reference | – | – | – |
| Female | 19482 | 1397 | 7.2 (6.8, 7.5) | 1.308 (1.213, 1.410) | < 0.001 | 1.275 (1.182, 1.375) | < 0.001 |
| **Age, years** | | | | | | | |
| 0-17 | 4252 | 450 | 10.6 (9.7, 11.6) | 1.722 (1.473, 2.012) | < 0.001 | 1.650 (1.409, 1.933) | < 0.001 |
| 18-59 | 40244 | 2268 | 5.6 (5.4, 5.9) | Reference | – | – | – |
| ≥ 60 | 2018 | 188 | 9.3 (8.1, 10.7) | 1.408 (1.335, 1.485) | < 0.001 | 1.195 (1.125, 1.269) | < 0.001 |
| **Geographical region** | | | | | | | |
| Guangzhou | 13528 | 497 | 3.7 (3.4, 4.0) | 0.721 (0.697, 0.747) | < 0.001 | 0.719 (0.694, 0.745) | < 0.001 |
| Shenzhen | 16475 | 1520 | 9.2 (8.8, 9.7) | Reference | – | – | – |
| Dongguan | 7359 | 379 | 5.2 (4.7, 5.7) | 0.534 (0.476, 0.600) | < 0.001 | 0.441 (0.390, 0.499) | < 0.001 |
| Other | 9185 | 510 | 5.6 (5.1, 6.0) | 0.781 (0.722, 0.801) | < 0.001 | 0.698 (0.661, 0.736) | < 0.001 |
| **Occupation** | | | | | | | |
| Students/Teachers | 2352 | 291 | 12.4 (11.1, 13.8) | 0.188 (0.161, 0.220) | < 0.001 | 0.187 (0.159, 0.219) | < 0.001 |
| Health care workers | 985 | 43 | 4.4 (3.2, 5.9) | 0.247 (0.210, 0.290) | < 0.001 | 0.226 (0.192, 0.266) | < 0.001 |
| Restaurant services | 397 | 76 | 19.1 (15.5, 23.4) | 0.681 (0.622, 0.745) | < 0.001 | 0.687 (0.627, 0.753) | < 0.001 |
| Unemployed/Home | 1579 | 677 | 42.9 (40.4, 45.4) | Reference | – | – | – |
| Workers | 6267 | 477 | 7.6 (7.0, 8.3) | 0.576 (0.556, 0.596) | < 0.001 | 0.606 (0.585, 0.628) | < 0.001 |
| Other | 34967 | 1342 | 3.8 (3.6, 4.1) | 0.556 (0.544, 0.569) | < 0.001 | 0.559 (0.546, 0.571) | < 0.001 |

*None: not vaccinated; part vaccination: < 14 days after first vaccination for viral vector (non-replicating) vaccine, after first vaccination or < 14 days after second vaccination for COVID-19 inactivated virus vaccine, and after first and second vaccination, or < 14 days after third vaccination COVID-19 protein subunit vaccine (if any); full vaccination: ≥ 14 days after first vaccination for viral vector (non-replicating) vaccine, ≥ 14 days after second vaccination for COVID-19 inactivated virus vaccine, ≥ 14 days after third vaccination for COVID-19 protein subunit vaccine, and < 7 days after booster vaccination (if any); booster vaccination: ≥ 7 days after second dose for COVID-19 viral vector (non-replicating) vaccines or ≥ 7 days after third dose for COVID-19 any vaccine (including protein subunit, inactivated virus, and viral vector [non-replicating] vaccines) (if any)
