# Supplementary material for: Effectiveness of booster vaccination with inactivated COVID-19 vaccines against SARS-CoV-2 Omicron BA.2 infection in Guangdong, China: a cohort study
Source: Front Immunol. 2023 Oct 17;14:1257360. doi: 10.3389/fimmu.2023.1257360 (PMC10616523; doi:10.3389/fimmu.2023.1257360)
Supplement: Supplementary file 6 [file DataSheet_6.docx]

**Supplementary Table 3** Characteristics of close contacts by inactivated virus vaccination status*****

| **Characteristics of close contacts** | **Full vaccination**  **(n = 10373****)** | **Booster vaccination (n =** **26727)** | **Overall**  **(n = 37100)** | ***P* value** |
| --- | --- | --- | --- | --- |
| **Age, years** | | | | |
| Median, IQR | 33 (26, 42) | 37 (29, 47) | 36 (28, 46) | < 0.001 |
| 18-29 | 3808 (36.7) | 6724 (25.2) | 10532 (28.4) | < 0.001 |
| 30-39 | 3464 (33.4) | 8429 (31.5) | 11893 (32.0) |  |
| 40-49 | 1621 (15.6) | 6502 (24.3) | 8123 (21.9) |  |
| 50-59 | 959 (9.3) | 4089 (15.3) | 5048 (13.6) |  |
| 60-69 | 521 (5.0) | 983 (3.7) | 1504 (4.1) |  |
| **Gender** | | | | |
| Male | 6232 (60.1) | 15410 (57.7) | 21642 (58.3) | < 0.001 |
| Female | 4141 (39.9) | 11317 (42.3) | 15458 (41.7) |  |
| **Geographical region** | | | | |
| Guangzhou | 2160 (20.8) | 9027 (33.8) | 11187 (30.2) | < 0.001 |
| Shenzhen | 4368 (42.1) | 9040 (33.8) | 13408 (36.1) |  |
| Dongguan | 2345 (22.6) | 3515 (13.2) | 5860 (15.8) |  |
| Other | 1500 (14.5) | 5145 (19.2) | 6645 (17.9) |  |
| **Occupation** | | | | |
| Students/Teachers | 125 (1.2) | 299 (1.1) | 424 (1.1) | < 0.001 |
| Health care workers | 99 (1.0) | 833 (3.1) | 932 (2.5) |  |
| Restaurant services | 99 (1.0) | 244 (0.9) | 343 (0.9) |  |
| Unemployed/Home | 291 (2.8) | 549 (2.1) | 840 (2.3) |  |
| Workers | 1746 (16.8) | 3691 (13.8) | 5437 (14.7) |  |
| Other | 8013 (77.2) | 21111 (79.0) | 29124 (78.5) |  |
| **Infection** | | | | |
| Yes | 712 (6.9) | 1419 (5.3) | 2131 (5.7) | < 0.001 |
| No | 9661 (93.1) | 25308 (94.7) | 34969 (94.3) |  |
| **Symptomatic COVID-19** | | | | |
| Yes | 508 (4.9) | 953 (3.6) | 1461 (3.8) | < 0.001 |
| No | 9865 (95.1) | 25774 (96.4) | 35639 (96.2) |  |
| **COVID-19 pneumonia** | | | | |
| Yes | 43 (0.4) | 75 (0.3) | 118 (0.3) | 0.040 |
| No | 10330 (99.6) | 26652 (99.7) | 36982 (99.7) |  |

Data are n (%), unless otherwise specified. *Full vaccination: ≥ 14 days after second vaccination (if any); booster vaccination: ≥ 7 days after third dose (if any).
