# Supplementary material for: Effectiveness of booster vaccination with inactivated COVID-19 vaccines against SARS-CoV-2 Omicron BA.2 infection in Guangdong, China: a cohort study
Source: Front Immunol. 2023 Oct 17;14:1257360. doi: 10.3389/fimmu.2023.1257360 (PMC10616523; doi:10.3389/fimmu.2023.1257360)
Supplement: Supplementary file 7 [file DataSheet_7.docx]

**Supplementary Table 4** The effectiveness of booster vaccination of COVID-19 inactivated vaccines against infection and COVID-19 illness caused by SARS-CoV-2 Omicron variant compared with full vaccination

| **Vaccination Status** | **Events/Participants (n/N, % [95% CI])** | | **Crude** | | **Adjusted*** | |
| --- | --- | --- | --- | --- | --- | --- |
|  | **Full vaccination^#^** | **Booster vaccination^&^** | **RR (95% CI)** | **VE (95% CI), %** | **RR (95% CI)** | **VE (95% CI), %** |
| **Infections** | | | | | | |
| Overall | 712/10373 (6.9 [6.4, 7.4]) | 1419/26727 (5.3 [5.1, 5.6]) | 0.761 (0.693, 0.835) | 23.9 (16.5, 30.7) | 0.714 (0.650, 0.784) | 28.6 (11.6, 35.0) |
| Age (years) | |  | | | | |
| 18-59 | 637/9852 (6.5 [6.0, 7.0]) | 1343/25744 (5.2 [5.0, 5.5]) | 0.796 (0.722, 0.878) | 20.4 (12.2, 27.8) | 0.739 (0.670, 0.816) | 26.1 (18.4, 33.0) |
| ≥ 60 | 75/521 (14.4 [11.6, 17.8]) | 76/983 (7.7 [6.2, 9.6]) | 0.498 (0.355, 0.699) | 50.2 (30.1, 64.5) | 0.501 (0.323, 0.777) | 49.9 (22.3, 67.7) |
| **Symptomatic COVID-19** | | | | | | |
| Overall | 508/10373 (4.9 [4.5, 5.3]) | 953/26727 (3.6 [3.4, 3.8]) | 0.718 (0.643, 0.802) | 28.2 (19.8, 35.7) | 0.604 (0.521, 0.700) | 39.6 (30.0, 47.9) |
| **Age (years)** | | | | | | |
| 18-59 | 455/9852 (4.6 [4.2, 5.1]) | 906/25744 (3.5 [3.3, 3.8]) | 0.753 (0.671, 0.845) | 24.7 (15.5, 32.9) | 0.643 (0.549, 0.752) | 35.7 (24.8, 45.1) |
| ≥ 60 | 53/521 (10.2 [7.8, 13.2]) | 47/983 (4.8 [3.6, 6.4]) | 0.443 (0.295, 0.667) | 55.7 (33.3, 70.5) | 0.374 (0.220, 0.638) | 62.6 (36.2, 78.0) |
| **COVID-19 pneumonia** | | | | | | |
| Overall | 43/10373 (0.4 [0.3, 0.6]) | 75/26727 (0.3 [0.2, 0.4]) | 0.676 (0.464, 0.984) | 32.4 (1.6, 53.6) | 0.478 (0.291, 0.787) | 52.2 (21.3, 70.9) |
| **Age (years)** | | | | | | |
| 18-59 | 37/9852 (0.4 [0.3, 0.5]) | 69/25744 (0.3 [0.2, 0.4]) | 0.713 (0.478, 1.064) | 28.7 (-6.4, 52.2) | 0.520 (0.302, 0.895) | 48.0 (10.5, 69.8) |
| ≥ 60 | 6/521 (1.2 [0.5, 2.6]) | 6/983 (0.6 [0.3, 1.4]) | 0.527 (0.169, 1.643) | 47.3 (-64.3, 83.1) | 0.162 (0.037, 0.709) | 83.8 (28.1, 96.3) |

RR = risk ratio; VE = vaccine effectiveness.

*Adjusted for sex, age, interval time of vaccination, occupation, and geographical region.

^#^Full vaccination: ≥ 14 days after second vaccination

^&^Booster vaccination: ≥ 7 days after third dose (if any).
